# Usefulness of an ultrathin endoscope for guidewire insertion during stent-in-stent placement

**DOI:** 10.1016/j.vgie.2023.07.017

**Published:** 2023-09-13

**Authors:** Takuro Hamada, Toshio Kuwai, Takeshi Mizumoto, Yuzuru Tamaru, Ryusaku Kusunoki, Sauid Ishaq, Hiroshi Kohno

**Affiliations:** 1 Department of Gastroenterology, National Hospital Organization, Kure Medical Center and Chugoku Cancer Center, Kure, Japan; 2 Gastroenterology Department, Russells Hall Hospital, Birmingham City University, Birmingham, United Kingdom; 1 Department of Gastroenterology, National Hospital Organization, Kure Medical Center and Chugoku Cancer Center, Kure, Japan

## Abstract

Video 1 Stent-in-stent placement using an ultrathin endoscope.

Stent-in-stent placement using an ultrathin endoscope.

Self-expandable metallic stent (SEMS) placement is commonly used to relieve malignant colonic obstructions.^1^, ^2^, ^3^ Despite its high clinical success rate, severe adverse events, including stent occlusion, may occur. Stent-in-stent placement is one possible solution; however, it is technically difficult and occasionally fails in difficult situations.^4^^,^^5^ In this video article, we discuss the usefulness of an ultrathin endoscope for guidewire insertion during stent-in-stent placement in a difficult situation.

The patient, a 68-year-old man, had an uncovered 22-mm × 12-cm SEMS placed for an obstruction caused by unresectable sigmoid colon cancer. Despite continuing chemotherapy, he complained of abdominal distension and constipation. Because a CT scan revealed stent occlusion due to tumor ingrowth, we decided to re-stent (Fig. 1).

**Figure 1:**
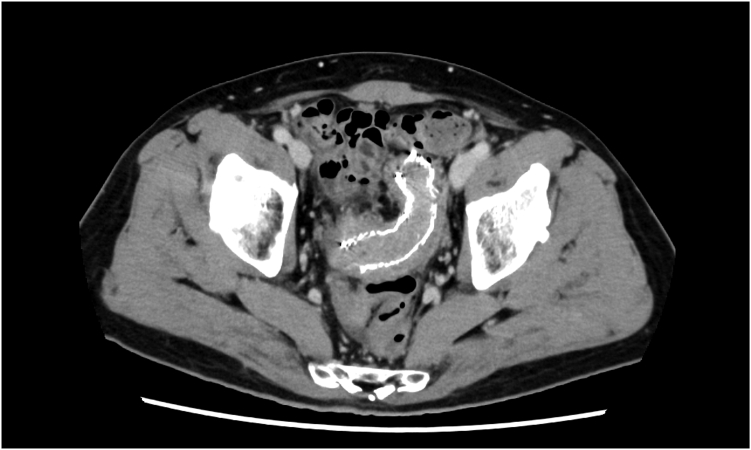
A CT scan revealed a colonic stent occlusion due to tumor ingrowth.

In addition, a colonoscopy revealed stent occlusion due to tumor ingrowth (Fig. 2). We attempted to pass a guidewire through the stenosis using a catheter, but it was difficult because of the previous stent’s strong flexion (Video 1, available online at www.videogie.org). Therefore, we switched the colonoscope to an ultrathin endoscope and attempted to insert it through the stent (Fig. 3A). The endoscope could be inserted approximately halfway through the stent. From this position, the guidewire was successfully passed through the stenosis using the through-the-scope technique under direct endoscopic and fluoroscopic guidance. We ensured that the guidewire was advanced within the lumen of the stent/stenosis and did not exit the lumen through a strut of an uncovered metal colonic stent under fluoroscopic guidance. The endoscope was removed while the wire remained in place; the guidewire was then brought out through the working channel of a large-caliber colonoscope, and the colonoscope was advanced over the guidewire (Fig. 3B). Two 22-mm × 12-cm SEMSs were successfully deployed to cover the entire stenosis (Fig. 4). No adverse events were noted, and oral intake started 3 days after the procedure. The patient was discharged 9 days later.

**Figure 2:**
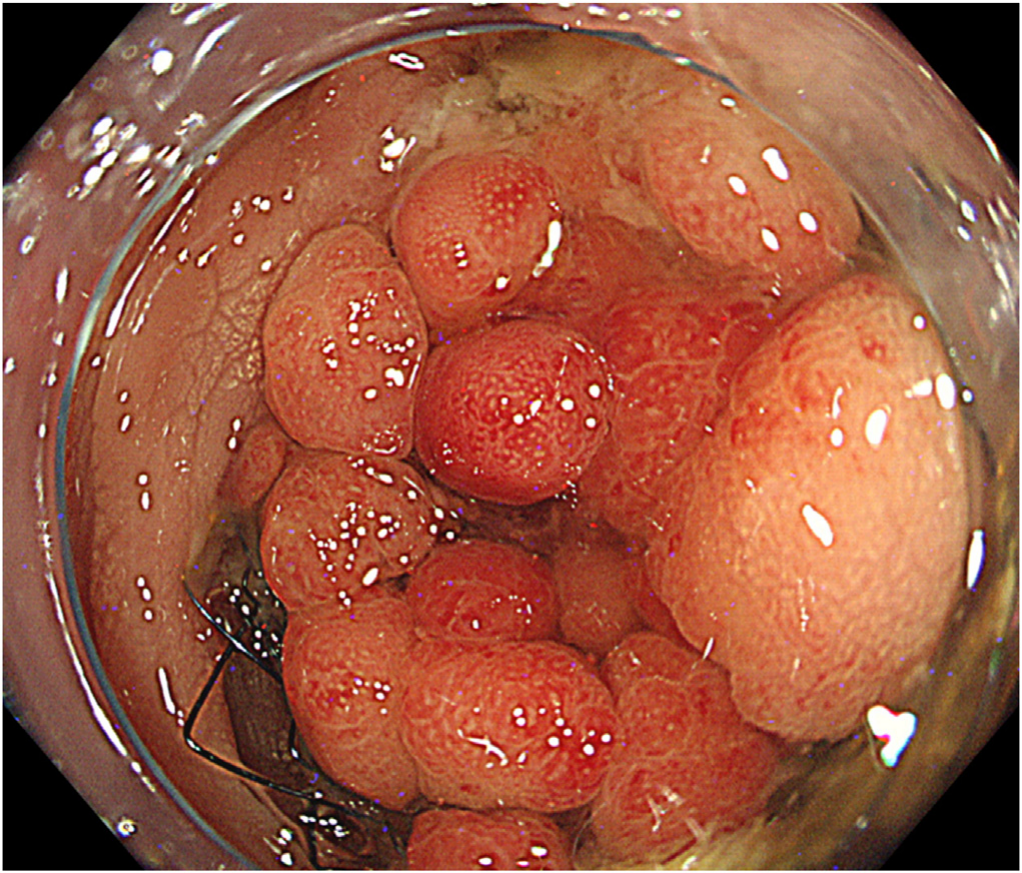
A colonoscopy revealed a stent occlusion due to sigmoid colon cancer.

**Figure 3:**
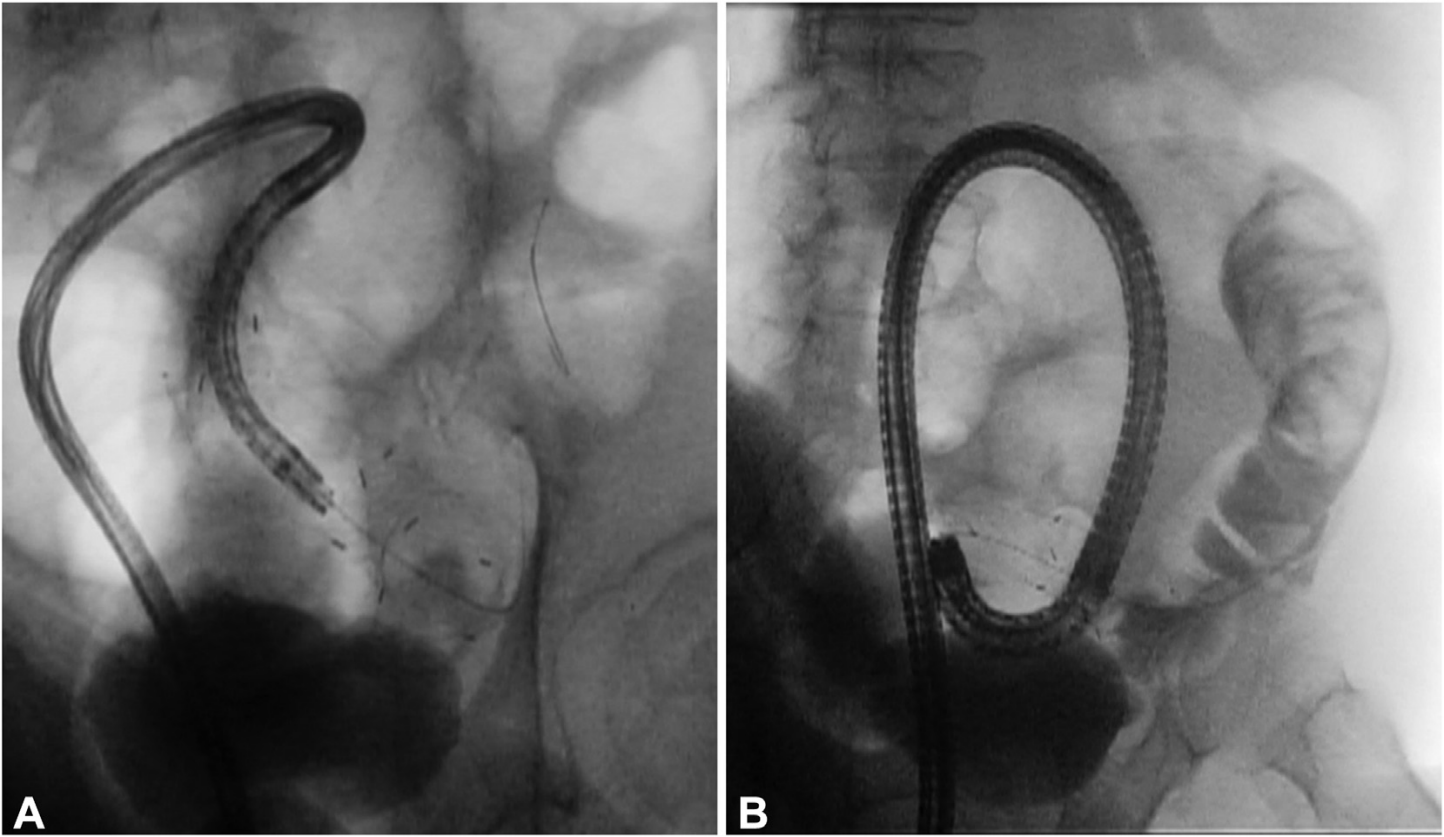
An ultrathin endoscope was inserted halfway through the stent. **A,** The guidewire was passed through the stenosis using the through-the-scope technique. **B,** A large-caliber colonoscope was reinserted over the guidewire.

**Figure 4:**
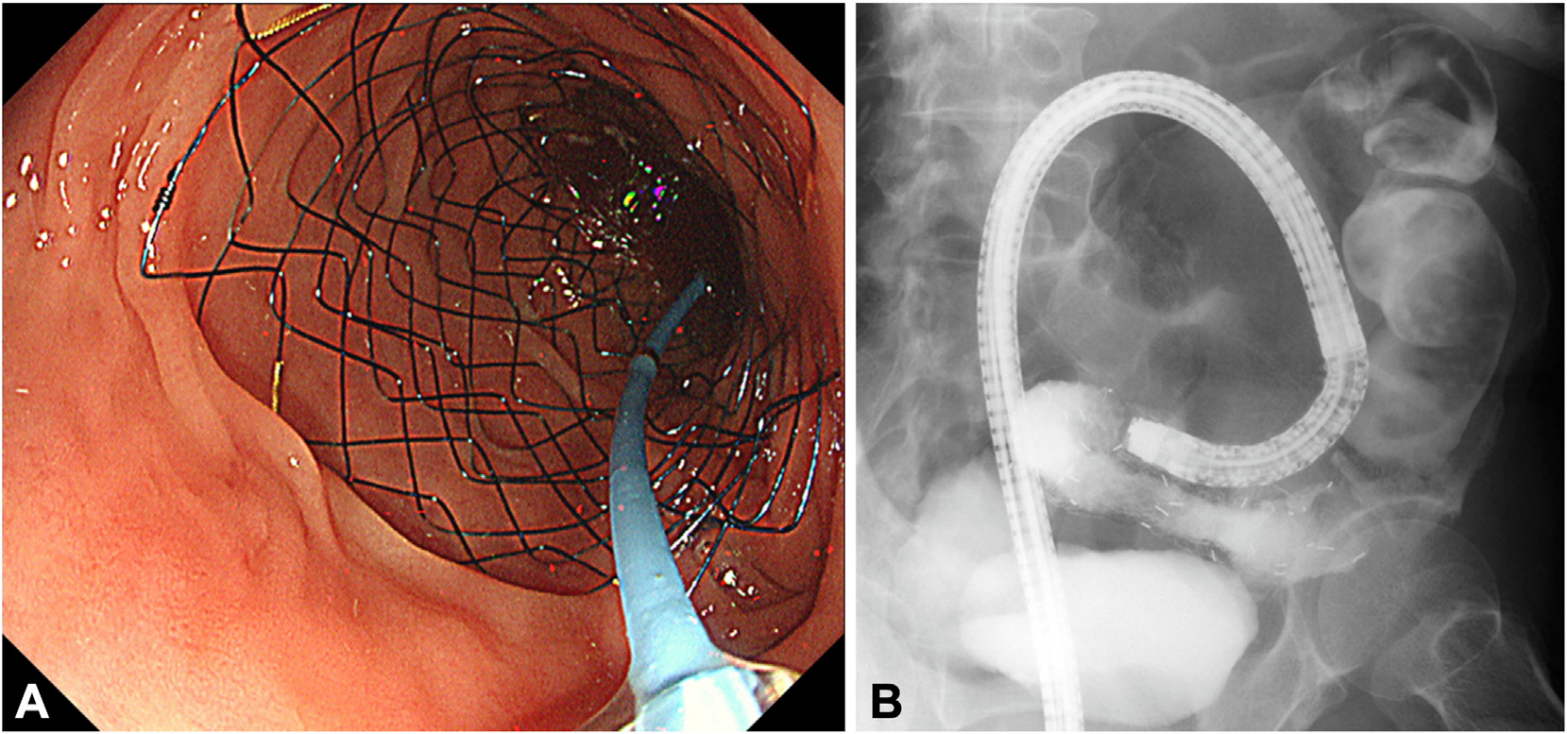
**A and B,** Two 22-mm × 12-cm self-expandable metallic stents were successfully placed.

This report demonstrates the usefulness of an ultrathin endoscope to traverse an area of stenosis to navigate a guidewire in stent occlusion with strong flexion, which is one of the most difficult cases for SEMS placement. This concept can be applied to challenging cases that are commonly reported as the so-called “over-the-catheter endoscope replacement” by Iboshi et al.^6^ For cases in the distal colon in particular, this concept would be more useful because it can help clinicians operate an ultrathin endoscope with favorable maneuverability. Additionally, Kobayashi et al^7^ reported that despite the presence of difficult cases in the proximal colon, this method can be performed by using an outer tube of a double-balloon endoscope (DBE). The procedure is as follows. A DBE device is inserted into the anorectal side of the stenosis. The DBE is then removed and the overtube is left inside with an inflated balloon. The overtube is cut at the area of the anus, and an ultrathin endoscope is inserted through the same. The endoscope is passed through the stenosis and removed with a guidewire in place. A SEMS can be subsequently deployed under fluoroscopic guidance. Therefore, this concept can be applied to special cases—including the present case—and common difficult cases for guidewire insertion at any location by devising it as described earlier. When using this method, the endoscopist should consider the possibility that adverse events, especially perforation, may occur. Thus, endoscopic manipulations must always be carefully performed.

In conclusion, an ultrathin endoscope can be used to navigate the guidewire in the right direction and to pass through the strong flexion, which could not have been overcome because of the stent occlusion caused by tumor ingrowth.

## Disclosure

The authors did not disclose any financial relationships.

## Supplementary data

Video 1 Stent-in-stent placement using an ultrathin endoscope.
